# Gauging the link between different views of aging facets and their associations with cognitive and mood functioning in midlife and older age: a cross-sectional study

**DOI:** 10.3389/fpsyg.2025.1676575

**Published:** 2025-10-29

**Authors:** Elena Carbone, Omar Paccagnella, Erika Borella

**Affiliations:** ^1^Department of General Psychology, University of Padova, Padova, Italy; ^2^Department of Statistical Sciences, University of Padova, Padova, Italy

**Keywords:** personal views of aging, subjective age, attitudes towards own aging, awareness of age-related change, working memory, mood

## Abstract

**Objectives:**

Personal Views of Aging (VoA) encompass different constructs capturing individuals' perceptions, attitudes, and expectations regarding their aging self, which are well-established influences of health-related and longevity outcomes over the adult life course. The present study aimed to investigate the associations among personal VoA facets, namely subjective age, Attitudes Toward Own Aging (ATOA), and Awareness of Age-Related Change (AARC), and their joint contribution in explaining mood and cognitive functioning in midlife and older age.

**Method:**

A sample of 350 community-dwelling individuals aged 40 to 92 years reported their mental felt age and completed the Attitudes Toward Own Aging scale (ATOA) and the Awareness of Age-Related Change questionnaire (AARC), assessing perceptions of age-related gains (AARC-Gains) and losses (AARC-Losses) in various functioning domains. They were also administered a working memory and a mood measure. Structural equation models were used to examine the associations between personal VoA, cognitive and mood outcomes.

**Results:**

Results revealed that AARC is more likely to act as a mediator between the global personal VoA facets and the mood outcome but not the cognitive one. Specifically, more positive ATOA scores were associated with high AARC-Gains and low AARC-Losses, and AARC-Losses were in turn associated with better mood functioning. Moreover, a youthful mental felt age was associated with higher ATOA and AARC-Gains scores, whereas greater AARC-Gains and AARC-Losses were associated with a poorer working memory performance.

**Discussion:**

These findings suggest a complex interplay between facets of personal VoA and further highlight their contributions to explaining particularly mood outcomes in midlife and older age.

## 1 Introduction

Personal Views of Aging (VoA) reflect individuals' perceptions, attitudes, and expectations regarding their aging self (Shrira et al., 2022). Extensive evidence demonstrates that personal VoA is related to psychological and physical health-related outcomes (Westerhof et al., 2023), and there is an emerging body of research linking personal VoA to cognitive functioning among older adults (Fernández-Ballbé et al., 2023).

If research has convincingly documented their implications for the promotion of a successful/healthy aging (Sabatini et al., 2025), most studies conducted so far have focused on single VoA facets in relation to relevant health-related and longevity outcomes (Shrira et al., 2022). Personal VoA is, in fact, an umbrella term encompassing a variety of related yet also sufficiently distinct constructs capturing the way individuals experience their aging (Brothers et al., 2017, 2019; Kornadt et al., 2020; Spuling et al., 2020), namely felt age, Attitudes Toward Own Aging (ATOA), and Awareness of Age-Related Change (AARC). Felt age, or the discrepancy between an individual's perceived age and actual chronological age, is seen as psychologically distancing oneself from one's “true” age and age peers in response to age-relevant experiences (Montepare, 2009). ATOA refers to individuals' general behavioral, cognitive, and affective evaluations of their aging process and their expectations about their experience of being older adults (Diehl et al., 2014). A youthful subjective age and positive ATOA are well-known predictors of various health-related and longevity indicators, including reduced depressive symptoms and better cognition (Debreczeni and Bailey, 2021; Fernández-Ballbé et al., 2023; Tully-Wilson et al., 2021). AARC captures two distinct subcomponents of aging-related daily-life experiences which can occur independently of one another, that is positive (AARC-Gains) and negative (AARC-Losses) subjective evaluations of aging-related experiences across domains of functioning (Brothers et al., 2019). Such a distinction is of interest given that holding AARC-Losses seems to have stronger and more consistent impact on physical and psychological outcomes than the protective effects of positive ones (Sabatini et al., 2020). When cognition is concerned, the few studies linking AARC to cognitive functioning reported mixed results, with some studies (Sabatini et al., 2021, 2023a; Testad et al., 2022) showing greater AARC-Gains and AARC-Losses associated with poorer cognitive performance and others (Sabatini et al., 2022; Voelkner and Caskie, 2023; Zhu and Neupert, 2020) showing limited associations between AARC and cognitive measures, with greater AARC-Losses but not AARC-Gains associated with poorer cognition.

What remains largely understudied, however, is the interplay among various personal VoA facets and their combined effects on psychological and cognitive measures. There are indeed conceptual differences among felt age, ATOA, and AARC, such as the fact that they capture global vs. behavior-specific evaluations (Brothers et al., 2017; Diehl et al., 2014). In particular, felt age and ATOA represent global evaluations of one's own aging process without an explicit reference to specific personal aging experiences. AARC, instead, is rooted in self-reflections and conscious awareness through which individuals appraise specific aging-related daily-life experiences in various domains of functioning (Brothers et al., 2017; Diehl et al., 2014; Miche et al., 2014). As a result, felt age and ATOA would more likely act as cognitive schema that prime individuals to notice and expect age-related changes in terms of gains and losses, thereby motivating or impeding behaviors that promote, in turn, health and wellbeing (Diehl and Wahl, 2010; Hummert, 2011). Empirical evidence, found in only one previous cross-sectional study to date (Brothers et al., 2017) at least to our knowledge, supports this notion: AARC was found to be more likely to mediate the associations between either felt age or ATOA, considered separately, and either self-rated functional health or wellbeing (life satisfaction) outcomes, with feeling older or holding more negative ATOA predicting particularly higher AARC-Losses and, in turn, poorer self-rated functional health or wellbeing.

This preliminary evidence set the stage for a closer understanding of the potential interplay among personal VoA constructs and of their combined impact on relevant psychological and cognitive outcomes, suggesting the need to jointly consider the specificity, multidimensionality, and valence characteristics of various facets of personal VoA, which remain worthwhile investigating issues.

The aims of the present cross-sectional study were therefore to further investigate: (i) the associations between global vs. behavior-specific personal VoA constructs, namely felt age, ATOA, and AARC; and (ii) the joint contribution of the VoA considered in explaining mood functioning, in terms of subclinical depressive symptoms, and cognitive functioning. This latter was specifically operationalized in terms of Working Memory (WM) performance, being considered a core cognitive mechanism sensitive to aging-related decline and accounting for age-related differences in various complex cognitive domains across the adult life span (Borella et al., 2008; Pezzuti et al., 2019), and only rarely examined in relation to personal VoA (Fernández-Ballbé et al., 2023).

In line with previous evidence (Brothers et al., 2017, 2019; Kornadt et al., 2020; Spuling et al., 2020), small-to-medium, significant associations between the personal VoA facets considered here could be expected, attesting to the fact that they represent different constructs.

Moreover, in line with previous reports (e.g., Brothers et al., 2017), AARC, reflecting perceptions of specific aging-related experiences, would more likely mediate the link between the more global facets of personal VoA (i.e., felt age and ATOA), mood and cognitive (WM) outcomes. To further confirm these notions, by means of mediation analyses, we tested and compared two models, one assuming that global personal VoA facets (i.e., felt age and ATOA) are associated with mood and cognitive outcomes via behavior-specific personal VoA (i.e., AARC-Gains and AARC-Losses) and the other assuming that it is behavior-specific personal VoA (i.e., AARC-Gains and AARC-Losses), to be associated with cognitive performance and mood functioning through global personal VoA facets (i.e., felt age and ATOA).

Based on previous evidence (e.g., Debreczeni and Bailey, 2021; Sabatini et al., 2020; Tully-Wilson et al., 2021), stronger associations between personal VoA measures and mood functioning compared to cognitive performance, and nuanced associations when the valence (positive vs. negative) of self-perceptions of aging is concerned in relation to the mood and cognitive outcomes examined, are hypothesized (e.g., Brothers et al., 2017; Sabatini et al., 2020).

The effects of chronological age along with gender, years of education, retirement, self-rated health and engaged lifestyle were considered in the analyses, given their associations with personal VoA (Sabatini et al., 2023c, 2025).

## 2 Methods

### 2.1 Participants

The study involved 350 community-dwelling, Italian native-speaker adults aged 40 to 92 years (59% females), recruited by word of mouth and volunteering for the study.

Inclusion criteria were as follows: (i) no history of major physical or mental health issues, assessed through a semi-structured interview (De Beni et al., 2008); (ii) a Montreal Cognitive Assessment-BLIND score ≥17 (MoCA-BLIND; Wittich et al., 2010)^1^; and (iii) a Geriatric Depression Scale score ≤ 5 Sheikh and Yesavage, (1986), i.e., no sign of neurocognitive disorder and major depressive symptoms.

Table 1 shows the descriptive statistics of participants' sociodemographic characteristics and measures of interest.

**Table 1:** Descriptive statistics of the socio-demographic characteristics and the measures of interest.

**Variable**		
Female (*n*, %)	208	59.43%
Chronological age (mean, SD)	62.04	10.83
Years of education (mean, SD)	12.31	3.99
Being retired (*n*, %)	144	41.14%
Engaged lifestyle (*n*, %)	305	87.14%
Self-rated health (mean, SD)	3.93	0.58
Montreal Cognitive Assessment-BLIND (mean, SD)	19.70	1.52
Backward digit span task (mean, SD)	6.92	3.52
Geriatric Depression Scale (mean, SD)	1.88	1.51
Mental felt age (mean, SD)	– 0.14	0.14
ATOA (mean, SD)	14.24	2.19
AARC-Gains (mean, SD)	18.87	3.19
AARC-Losses (mean, SD)	11.33	3.55

ATOA, attitudes toward own aging; AARC, awareness of age-related change.

### 2.2 Materials

#### 2.2.1 Personal views of aging

*Mental felt age*. Participants were asked to provide their mental felt age with a single-item question: “*Do you feel mentally younger, the same, or older than your real age? Please indicate the mental age that you feel*”. Proportional discrepancy scores (dependent variable) were calculated for each participant as a measure of mental felt age (Debreczeni and Bailey, 2021) as follows: mental felt age —chronological age/chronological age, with negative scores corresponding to feeling mentally younger than one's own chronological age.

*Attitudes Toward Own Aging* (ATOA; adapted from Lawton, 1975). It consists of 5 items assessing individuals' self-perceptions of aging. Participants rated each item on a 4-point Likert scale (from 1 = completely disagree to 4 = completely agree). The dependent variable was obtained by summing the scores on each item (max = 20; α = 0.81; current sample: ω = 0.61),^2^ with higher scores corresponding to more positive attitudes toward own aging.

*Awareness of Age-Related Change (*AARC; adapted from Kaspar et al., 2019). This scale comprises 10 items, 5 assessing AARC-Gains and 5 assessing AARC-Losses. Out of the 5 items in each scale, there is 1 item for each of the AARC life and behavioral domains (health/physical functioning, cognitive functioning, interpersonal relationships, socio-cognitive and socio-emotional functioning, lifestyle engagement). Participants rated how much each item applied to them on a 5-point Likert scale (from 1 = not at all to 5 = very much). The dependent variables were the scores for AARC-Gains and AARC-Losses, calculated by summing the 5 items falling into the respective subscales (max = 25; AARC-Gains ω = 0.72; AARC-Losses ω = 0.80; current sample: AARC-Gains ω = 0.60; AARC-Losses ω = 0.71) (see text footnote 2). Higher scores indicate greater AARC-Gains and AARC-Losses, respectively.

#### 2.2.2 Cognitive functioning

*Backward digit span task* (De Beni et al., 2008). This WM measure (see Borella et al., 2020; Pezzuti et al., 2019) involves presenting series of digits (1s per digit), and participants had to repeat each series in the backward order. The series started from two digits and rose to eight, each level containing two strings of digits. The task ended when the participant failed to correctly repeat two strings of the same length. One point was assigned for each sequence correctly recalled. The dependent variable was the total number of correct trials recalled (max = 14).

#### 2.2.3 Mood functioning

*Geriatric Depression Scale* (GDS; Sheikh and Yesavage, 1986). This short version of the Geriatric Depression Scale is a 15-item self-report questionnaire developed to assess depression among older adults. Responses are recorded on a dichotomous “yes-no” scale, with 5 items exploring positive attitude toward life (e.g., satisfied with life, full of energy) and 10 items assessing dissatisfaction with life (e.g., feel helpless, hopeless, bored, worried about the future) or personal issues (e.g., memory concerns, dropped activities/interests). Every item contributes 1 point to the final score, with scores greater than 5 considered as indicative of mild-to-severe depression (Sheikh and Yesavage, 1986).

In the current study, the GDS was used both as a screening measure for assessing inclusion criteria and as a measure of mood functioning (max = 5).

#### 2.2.4 Covariates

Gender (0 = male, 1 = female), chronological age, years of education, being retired (0 = no, 1 = yes), self-rated health and engaged lifestyle (0 = no free time activities, 1 = involvement in leisure or physical activities) were used as covariates, due to their associations with personal VoA (Sabatini et al., 2023c, 2025). As for self-rated health, participants were asked to rate their physical and psychological health on a 5-point Likert scale (1 = *very poor*; 5 = *very good*) with two *ad-hoc* questions (“*How do you rate your overall physical health?*” and “*How do you rate your overall psychological health?*”). A composite score expressing overall self-rated health was calculated and considered, with higher scores corresponding to better perceived health.

### 2.3 Procedure

Participants were contacted by phone to complete an individual interview of about 60 mins with a trained experimenter. Participants were asked to sit in a quiet area of their home to avoid hearing issues. After obtaining their consent, the experimenter guided participants through the completion of tasks and questionnaires, ensuring that they were able to hear and understand the instructions and stimuli clearly, as following: a semi-structured interview assessing demographic characteristics, physical and mental health status and felt age; the MOCA-BLIND, the Backward Digit Span task, the AARC; the ATOA and the GDS.

### 2.4 Analytical strategy

Structural Equation Modeling (SEM) on observed variables (Kline, 2016) was adopted to estimate the relationships among VoA facets (ATOA, mental felt age, AARC-Gains, AARC-Losses) and cognitive (WM performance) and psychological (mood) functioning.

In particular, according to extant theoretical framework and previous evidence (Brothers et al., 2017; Shrira et al., 2022), we tested: (i) a first model (Model A) assuming that mental felt age and ATOA (acting as “cognitive schema” that prime individuals to notice and expect age-related changes) are associated with WM performance (backward digit span) and mood (GDS), both directly and indirectly through AARC-Gains and AARC-Losses; and (ii) a second model (Model B) assuming that AARC-Gains and AARC-Losses are associated with cognitive and mood functioning both directly and indirectly through mental felt age and ATOA.

Chronological age along with gender, years of education, retirement, self-rated health and engaged lifestyle were used as covariates. Covariances between ATOA and mental felt age, AARC-Gains and AARC-Losses and GDS and backward digit span were specified. AARC-Gains and AARC-Losses were assumed to have the same variance.

A model comparison approach was used to identify the best model, calculating and evaluating several goodness of fit statistic (likelihood ratio test, RMSEA, CFI, TFI and SRMR).

Models were run by using the Stata software (StataCorp, 2023).

## 3 Results

**Figure 1:**
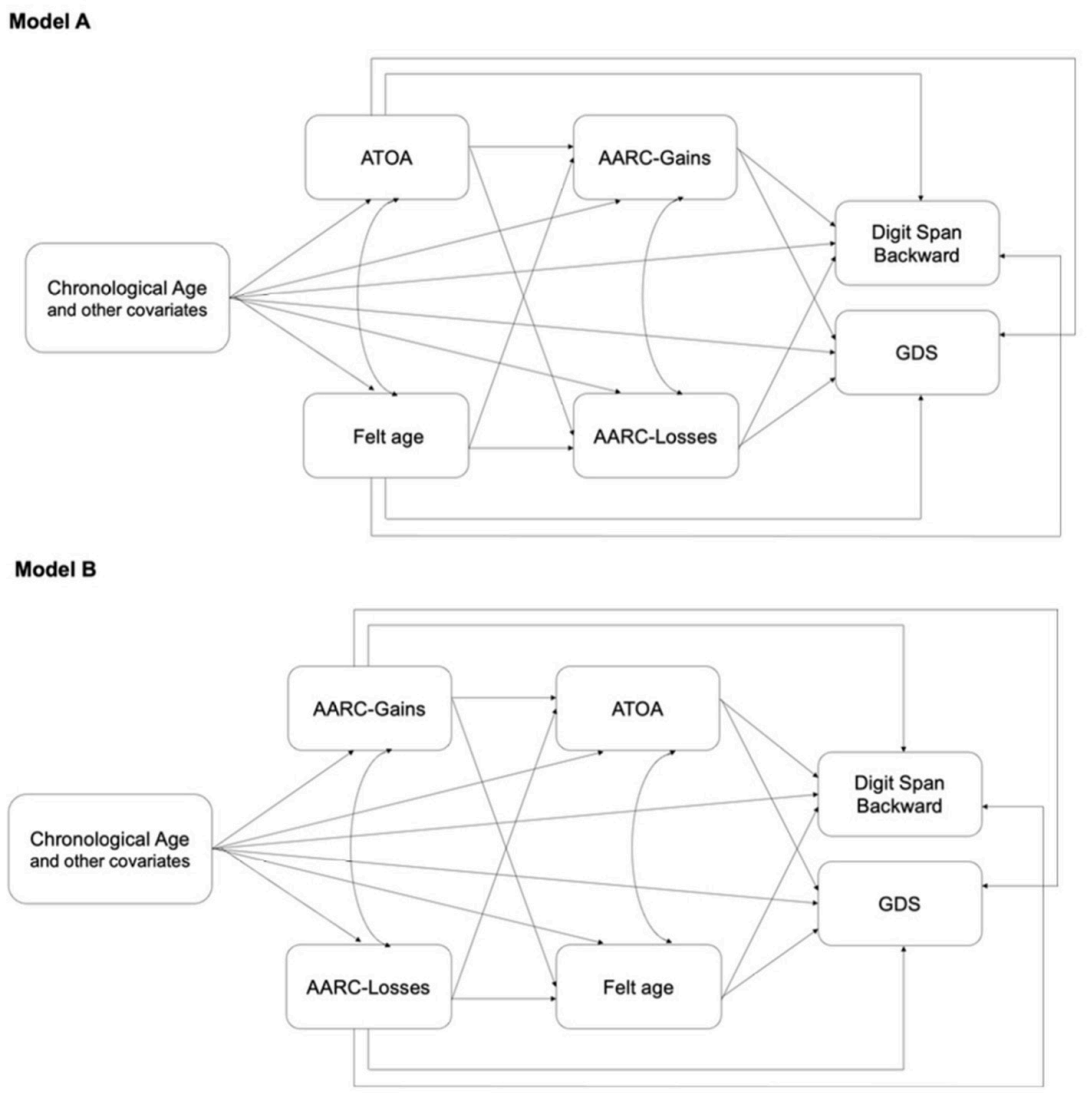
Graphical representation of the two SEM models tested. ATOA, attitudes toward own aging; AARC, awareness of age-related change; GDS, geriatric depression scale. Model A assumes that mental felt age and ATOA are associated with cognitive (backward digit span) and mood (GDS) functioning, both directly and indirectly through AARC-Gains and AARC-Losses, whereas Model B assumes that AARC-Gains and AARC-Losses are associated with cognitive and mood functioning both directly and indirectly through mental felt age and ATOA.

The two SEM models were initially estimated, but the estimated covariance between GDS and backward digit span was not statistically significant. Therefore, the models were specified again without such covariance (see Figure 1 for a graphical representation of the two estimated models).

Table 2 reports goodness of fit indicators of the final estimated models. All indicators support a very good model fit, and were slightly better for Model A than for Model B. Therefore, also accounting for extant theoretical framework and previous evidence (Brothers et al., 2017; Shrira et al., 2022), Model A was assumed to be the best model.^3^

**Table 2:** Comparison of the two model fit indices.

**Fit indicator**	**Model A**	**Model B**
χ^2^	2.835 (df = 2; *p*-value = 0.242)	3.571 (df = 2; *p*-value = 0.168)
RMSEA	0.035	0.047
CFI	0.998	0.996
TLI	0.949	0.904
SRMR	0.008	0.011
Coefficient of determination	0.471	0.473
AIC	11,832.231	11,832.967
BIC	12,067.565	12,068.301
Log likelihood	– 5,855.12	– 5,855.49

**Figure 2:**
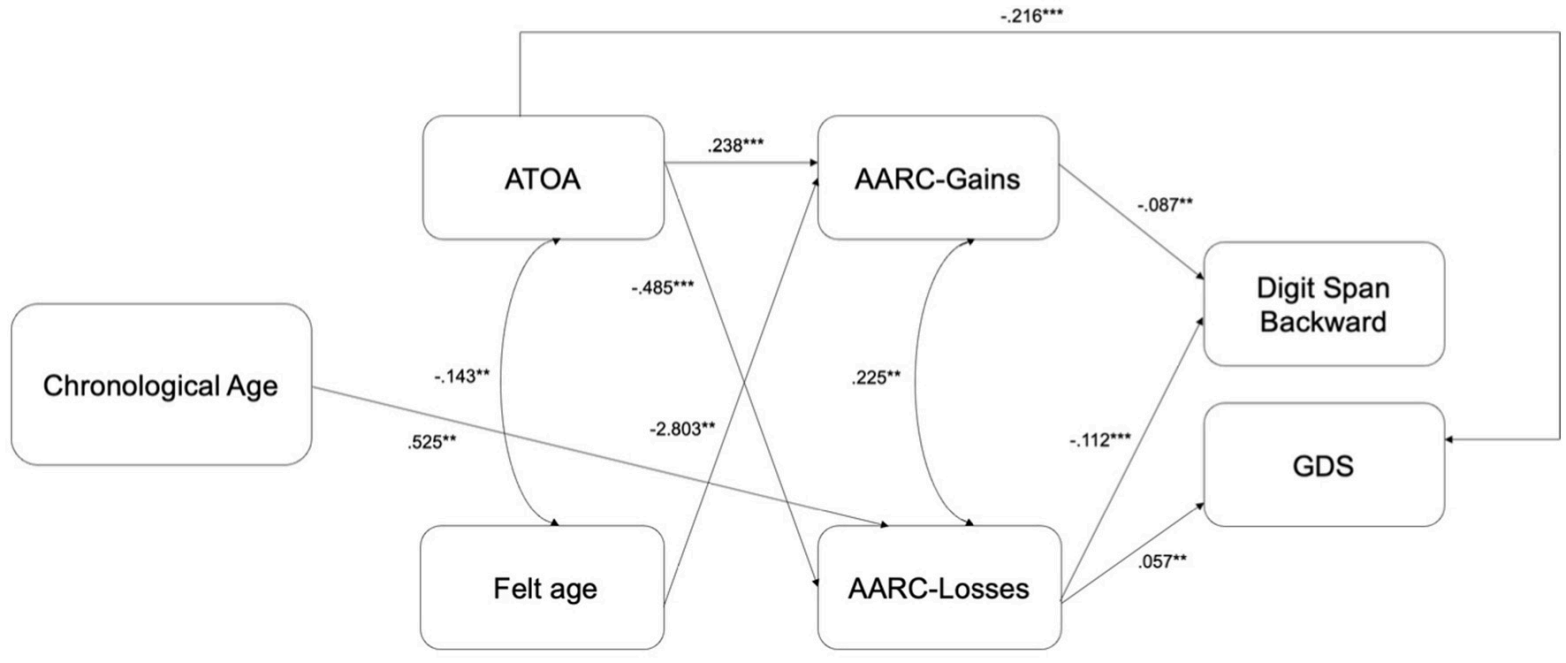
Results of the best model. ATOA, attitudes toward own aging; AARC, awareness of age-related change; GDS, geriatric depression scale. Significant total effects (standardized solutions) are reported. *** p < 0.01; ** p < 0.05.

Figure 2 shows the main results according to the final model: statistically significant total effects are highlighted and reported. All model estimates, decomposed into direct, indirect and total effects, are reported in Supplementary Table 1. Standardized estimates are computed.

Results showed a significant, negative covariance between ATOA and mental felt age, suggesting that people feeling mentally younger than their chronological age reported more positive attitudes toward own aging (see Figure 2 and Supplementary Table 1). A significant, positive covariance between AARC-Gains and AARC-Losses also emerged, with individuals who reported greater AARC-Gains also reporting greater AARC-Losses (see Figure 2 and Supplementary Table 1).

The effects (both direct and total) of ATOA on AARC-Gains and AARC-Losses were significant and positive for the former and negative for the latter, respectively, suggesting that individuals who reported more positive attitudes toward own aging also displayed greater AARC-Gains and a lower AARC-Losses (see Figure 2 and Supplementary Table 1). The effects (both direct and total) of mental felt age were significant and negative only on AARC-Gains, whereby individuals feeling younger than their chronological age also reported greater AARC-Gains (see Figure 2 and Supplementary Table 1).

The (direct and total) effects of both AARC-Gains and AARC-Losses on the backward digit span task were significant and negative, suggesting that individuals scoring higher on this task reported lower awareness of age-related gains and losses (see Figure 2 and Supplementary Table 1). The ATOA showed a negative significant direct effect on the backward digit span task, but such an effect was no longer significant totally (see Figure 2 and Supplementary Table 1).

Results also revealed significant (direct and total) effects of ATOA and AARC-Losses, but not AARC-Gains, on GDS scores, which were negative for the former and positive for the latter, thereby suggesting that more positive attitudes toward own aging were associated with lower depressive symptoms, whereas a greater awareness of age-related losses with greater depressive symptoms (see Figure 2 and Supplementary Table 1).

Concerning indirect effects, results revealed a significant, negative indirect effect, through AARC, of ATOA on GDS, but not on backward digit span (see Supplementary Table 1). No indirect effects emerged for mental felt age on backward digit span or on GDS.

As for the covariates, chronological age showed a total, significant and positive effect on AARC-Losses, and an indirect, positive effect on GDS only (see Supplementary Table 1).^4^ Gender showed significant, direct and total positive effects on AARC-Gains and GDS scores (see Supplementary Table 1). Self-rated health had direct, indirect and total effects on almost all equations except for the backward digit span task (see Supplementary Table 1). Education showed direct and total positive effects on ATOA scores and backward digit span task, indirect and total negative effects on AARC-Losses scores as well as an indirect negative effect on GDS scores (see Supplementary Table 1). An engaged lifestyle had direct and total, but not indirect, effects on all equations except for mental felt age, GDS and backward digit span task (see Supplementary Table 1). Being retired was associated (direct and total effects) only with greater AARC-Gains scores (see Supplementary Table 1).

## 4 Discussion

In this cross-sectional study, we thoroughly examined associations between global vs. behavior-specific personal VoA facets (i.e., subjective age, ATOA, and AARC-Gains and AARC-Losses) and their joint contribution in explaining working memory performance and mood functioning in a sample of middle-aged and older adults.

### 4.1 Associations between different personal VoA dimensions

In line with previous evidence (Brothers et al., 2017, 2019; Kornadt et al., 2020; Spuling et al., 2020), our results showed small-to-medium, significant associations between the personal VoA facets considered here: A more positive ATOA was associated with a youthful mental felt age, greater AARC-Gains, and lower AARC-Losses; a youthful mental felt age was associated with higher AARC-Gains only; and the two AARC facets were positively associated with each other. Such a pattern of results further confirms that such personal VoA facets are associated yet reflect different constructs. It also further highlights the importance of considering the coexistence of perceived gains and losses in various domains of functioning when assessing self-perceptions of aging among the middle-aged and older adult population (Brothers et al., 2017, 2019; Sabatini et al., 2021).

### 4.2 Effects of personal VoA on mood functioning

In line with the only study (Brothers et al., 2017) conducted to examine, though separately, the associations between personal VoA facets and functional health and wellbeing outcomes, our results showed that behavior-specific personal VoA, i.e., AARC, more likely mediates the associations between global personal VoA (i.e., ATOA) and health-related outcomes (i.e., mood). In particular, ATOA had a direct effect on mood (GDS). Of more interest, an indirect effect of ATOA, through AARC, on mood (GDS) emerged whereby more positive ATOA, leading to lower AARC-Losses, explain, in turn, lower signs of depression. Our results align with extant theoretical accounts framing VoA in relation to relevant health-related outcomes over the life course (see Levy, 2009; Shrira et al., 2022) and seem to further suggest how global evaluations of one's own aging process, particularly ATOA, more likely prime self-reflections and conscious awareness of aging-related daily-life behaviors and experiences in various domains of functioning, captured by AARC, and impact, in turn, particularly psychological functioning (Brothers et al., 2017). Moreover, the fact that negative constructions of one's aging experience (AARC-Losses) seem more relevant than positive ones (AARC-Gains) in impacting psychological outcomes when depressive symptoms, as done here, are concerned, aligns with previous evidence showing the former consistently linked to poorer emotional wellbeing and the latter more likely linked to life satisfaction or psychological wellbeing than to depressive symptoms (Sabatini et al., 2020).

### 4.3 Effects of personal VoA on cognition

Our results also further highlight, as expected and in line with previous evidence (e.g., Debreczeni and Bailey, 2021; Sabatini et al., 2020; Tully-Wilson et al., 2021), that personal VoA might more likely be related to psychological functioning than to cognition. Associations between VoA and cognitive outcomes are in fact usually mixed compared with those between VoA and mental health/psychological functioning (e.g., Sabatini et al., 2020; Tully-Wilson et al., 2021). Nonetheless, and interestingly, greater AARC-Gains and AARC-Losses were associated with worse working memory performance. This aligns with previous studies (Sabatini et al., 2021, 2023b) and could suggest that a greater conscious experience, perception, and awareness of changes accompanying aging, of positive and negative valence, impact cognitive performance in tasks, as used here, requiring the use of attentional/processing resources. Our results also seem to suggest that where cognitive outcomes, or at least WM performance are concerned, AARC plays a greater role than other personal VoA facets, such as felt age and ATOA, possibly because AARC captures dynamic self-evaluations rooted in a person's everyday life (Miche et al., 2014) and could therefore be more salient when individuals face a cognitive task than more global and overall perceptions of aging that are not necessarily linked to specific experiences (Hess, 2006; Lawton, 1975; Miche et al., 2014). A closer understanding of the psychological pathways (e.g., metacognitive processes, sense of control, motivation, self-efficacy, coping strategies) linking VoA to cognition would help clarify such a complex relationship (Zhu and Neupert, 2020).

It is worth mentioning that, in contrast to previous findings (e.g., Brothers et al., 2017; Debreczeni and Bailey, 2021), we found little to no direct or indirect associations between felt age and both other personal VoA facets and cognitive and mood outcomes. Such a result might lie in the nuanced aspect of felt age considered (mental felt age instead of the commonly used broader discrepancy between how old individuals feel compared with their chronological age) and in having jointly assessed the interplay between global VoA facets [i.e., felt age and ATOA, which were examined separately in Brothers et al. (2017)], AARC, and cognitive and mood functioning. Further studies are therefore needed to better understand the role of various dimensions of felt age (Diehl et al., 2014) in relation to other nuances of VoA and relevant health-related outcomes over the adult life span.

It should be noted that an older chronological age was associated with greater AARC-Losses and, indirectly through VoA facets, signs of depression, further suggesting that accumulating stressful life or health-related events with increasing age might exacerbate individuals' self-perceptions of age-related losses and impact mood functioning (Rupprecht et al., 2022; Sabatini et al., 2022, 2023c).

The associations between personal VoA and WM performance was not affected by age. Such a result is in contrast with previous evidence of differential VoA-cognition associations depending on age group (e.g., Sabatini et al., 2021; Siebert et al., 2020). Nonetheless, the discrepancy between our and other studies might lie in that different personal VoA facets (e.g., AARC-Gains and AARC-Losses specifically examined using only the cognitive domain items in Sabatini et al., 2021) or different cognitive domains (e.g., a comprehensive index of reasoning, WM and processing speed tasks in Siebert et al., (2020); memory measures in Sabatini et al., 2021) were considered in relation to chronological age, which makes it difficult to draw conclusions. Further studies are therefore needed to better capture potential differential associations between different VoA dimensions and cognitive mechanisms and their complex interplay as a function of the life stage considered.

In line with the literature (e.g., Sabatini et al., 2023a, 2025), other sociodemographic characteristics, lifestyle, and self-rated health also helped explain personal VoA facets and cognitive -WM- and mood outcomes considered.

In particular, being female was associated with higher signs of depression, in line with previous evidence (Girgus et al., 2017), and with greater AARC-Gains, as previously found (Testad et al., 2022), possibly due to the different cultural influences, expectations, and life experiences men and women are exposed to, which are then reflected in their self-perceptions of their aging (Kornadt et al., 2020; Sabatini et al., 2023b). Self-rated health was also found to be associated with almost all the outcomes considered except cognitive functioning, further confirming the well-known link between better self-rated health and positive VoA (Debreczeni and Bailey, 2021; Sabatini et al., 2020; Tully-Wilson et al., 2021) as well as better mood functioning (Chang-Quan et al., 2010) in midlife and older age.

Educational attainment was, then, linked with better cognitive performance and fewer signs of depression, in line with previous evidence (Borella et al., 2023), whereas educational attainment and an engaged lifestyle, in terms of participation in leisure and physical activities, were found to be associated with all personal VoA facets except AARC-Gains for the former and mental felt age for the latter, in a positive vein. Such a pattern of findings confirms that educational attainment is a protective factor for cognitive and mood functioning in midlife and older age (Borella et al., 2023). Furthermore, these results suggest that individuals' lifestyle habits and life experiences, being well-known proxies for cognitive reserve and thus prompting a successful/healthy aging through an “enriched and stimulating environment” (Borella et al., 2023), might have a nuanced role in eliciting positive evaluations and self-perceptions of one's aging process and aging-related changes, depending on the VoA facet considered (Sabatini et al., 2020, 2023c). Because it has been acknowledged that behavioral pathways (e.g., health-enhancing and adaptive behaviors) could link VoA to individuals' health-related outcomes (Sabatini et al., 2025; Westerhof et al., 2023), a closer and more comprehensive understanding of relevant proxies of cognitive reserve in relation to VoA and cognitive and psychological functioning would allow for clarification of such a complex interplay. Finally, being retired was associated with greater AARC-Gains. In line with previous evidence (Sabatini et al., 2020), this result might stem from the fact that AARC-Gains reflect self-perceptions of age-related changes (e.g., having more time to pay attention to one's health and interests and to appreciate meaningful relationships) more likely experienced and appraised when individuals are no longer engaged in a job.

## 5 Limitations

Despite such interesting results, some limitations should be acknowledged. First, although our results highlight that AARC more likely links global evaluations of one's aging process, captured particularly by ATOA, to mood outcomes, the pattern of associations that emerged is modest. Moreover, the cross-sectional nature of our study prevents clear conclusions on the causal or bidirectional relationships among VoA facets and between them and cognitive (WM) performance and mood functioning. Therefore, the present study can be considered a first attempt to analyze various VoAs in relation to cognitive and psychological domains. However, studies adopting more comprehensive and robust measures of personal VoA, as recommended by (Sella et al. 2025), and large batteries of cognitive tasks to better capture their interplay with health-related outcomes over the life course are needed. Researchers should also attempt to include generalized VoA (e.g., old age stereotypes, essentialism of aging), thought to influence personal VoA which was not done here. Moreover, our study involved a sample of cognitively healthy, well-educated, and actively engaged adults and older adults, so the results might not be generalizable to the wider population; therefore, further studies should attempt to confirm and expand our results in more heterogeneous samples in terms of sociodemographic characteristics and health status.

## 6 Conclusions

Our findings suggest a complex interplay between different personal VoA and their contributions to explaining mood functioning and cognitive, at least working memory, performance in midlife and older age, above and beyond other relevant sources of individual differences related to socio-demographic, health status and lifestyle habits characteristics. A closer understanding of the associations among facets of such a multidimensional concept as VoA and their joint or differential role in shaping health-related outcomes, including cognitive and psychological ones, over the adult life course is paramount not only from a theoretical perspective but also from a clinical/applied viewpoint. Such a clarification would be of interest to detect, by more comprehensively assessing VoA, those individuals at risk of developing negative VoA and to design intervention approaches capable of addressing and mitigating key views of one's aging process toward the promotion of healthy aging.

## Data Availability

The raw data supporting the conclusions of this article will be made available by the authors, without undue reservation.

## Footnotes

^1^The 17-point cut-off, adjusted for educational attainment (i.e., with the additional point for individuals with fewer than 12 years of education), was chosen considering the absolute shift calculation provided by (Wittich et al. 2010), whereby the cut-off value for the MOCA-BLIND was reduced by the absolute value of points (i.e., 8) that are omitted from the full version of the MOCA.

^2^Reliability indicators are in line with those reported in previous studies (for ATOA see Tully-Wilson et al., 2021, and also the systematic review by Sella et al., 2025; for AARC, see Schlomann et al., 2022; Schönstein et al., 2023; Sabatini et al., 2023b; Kaspar et al., 2022). It is to note that the somewhat lower item correlations for the AARC-Gains scale reflect the relatively brief nature of the scale (5 items) and the purposeful use of heterogeneous items that sample across the construct space (see Kaspar et al., (2022).

^3^We run two separate models for backward digit span test and GDS, but results did not change with respect to the model comprising both backward digit span and GDS. Therefore, also for sake of parsimony, we chose to define and estimate a single model.

^4^We estimated the same model by including age as a categorical variable considering more faceted age ranges [adult: 40-50; middle age: 51-65; early old age: 66-74; old age: 75+] or testing non-linear effects for chronological age results do not change, but no statistically significant relationship with age emerged for backward digit span as well as ATOA, AARC-Gains, AARC-Losses, and GDS.
