# Supplementary material for: Gauging the link between different views of aging facets and their associations with cognitive and mood functioning in midlife and older age: a cross-sectional study
Source: Front Psychol. 2025 Oct 29;16:1676575. doi: 10.3389/fpsyg.2025.1676575 (PMC12605007; doi:10.3389/fpsyg.2025.1676575)
Supplement: Supplementary file 1 [file Table_1.docx]

**Supplemental materials**

Gauging the link between different Views of Aging facets and their associations with cognitive and mood functioning in midlife and older age: A cross-sectional study.

Table S1. Estimates of the best model (Model A), disaggregated by direct, indirect and total effects. Standardized estimates are reported.

|  | **Direct** | **Indirect** | **Total** |
| --- | --- | --- | --- |
| *Mental felt age* |  |  |  |
| Chronological age | – 0.008 | – | – 0.008 |
| Being female | – 0.009 | – | – 0.009 |
| Years of education | 0.001 | – | 0.001 |
| Self-rated health | – 0.050 ** | – | – 0.050 ** |
| Being retired | 0.012 | – | 0.012 |
| Engaged lifestyle | – 0.008 | – | – 0.008 |
|  |  |  |  |
| *ATOA* |  |  |  |
| Chronological age | – 0.290 | – | – 0.290 |
| Being female | – 0.287 | – | – 0.287 |
| Years of education | 0.096 ** | – | 0.096 ** |
| Self-rated health | 1.208 ** | – | 1.208 ** |
| Retired | 0.404 | – | 0.404 |
| Engaged lifestyle | 0.672 * | – | 0.672 * |
|  |  |  |  |
| *AARC-Gains* |  |  |  |
| Mental felt age | – 2.803 * | – | – 2.803 * |
| ATOA | 0.238 ** | – | 0.238 ** |
| Chronological age | – 0.008 | – 0.046 | – 0.054 |
| Being female | 0.809 * | – 0.042 | 0.767 * |
| Years of education | – 0.067 | 0.022 | – 0.045 |
| Self-rated health | 0.216 | 0.428 ** | 0.644 * |
| Retired | 1.049 * | 0.064 | 1.113 * |
| Engaged lifestyle | 1.490 ** | 0.183 | 1.673 ** |
|  |  |  |  |
| *AARC-Losses* |  |  |  |
| Mental felt age | 1.097 | – | 1.097 |
| ATOA | – 0.485 ** | – | – 0.485 ** |
| Chronological age | 0.393 | 0.132 | 0.525 * |
| Being female | 0.112 | 0.129 | 0.241 |
| Years of education | – 0.054 | – 0.046 ** | – 0.100 * |
| Self-rated health | – 1.203 ** | – 0.641 ** | – 1.845 ** |
| Retired | – 0.022 | – 0.183 | – 0.206 |
| Engaged lifestyle | – 1.021 * | – 0.335 * | – 1.356 ** |
|  |  |  |  |
| *Geriatric Depression Scale (GDS)* |  |  |  |
| AARC-Gains | – 0.020 | – | – 0.020 |
| AARC-Losses | 0.057 * | – | 0.057 * |
| Mental felt age | 0.168 | 0.120 | 0.288 |
| ATOA | – 0.216 ** | – 0.033 * | – 0.249 ** |
| Chronological age | 0.007 | 0.093 * | 0.100 |
| Being female | 0.725 ** | 0.059 | 0.783 ** |
| Years of education | 0.007 | – 0.026 ** | – 0.018 |
| Self-rated health | – 0.414 ** | – 0.389 ** | – 0.802 ** |
| Retired | – 0.170 | – 0.120 | – 0.290 |
| Engaged lifestyle | 0.070 | – 0.259 ** | – 0.189 |
|  |  |  |  |
| *Backward Digit Span task* |  |  |  |
| AARC-Gains | – 0.087 * | – | – 0.087 * |
| AARC-Losses | – 0.112 ** | – | – 0.112 ** |
| Mental felt age | – 1.379 | 0.120 | – 1.259 |
| ATOA | – 0.135 * | 0.034 | – 0.101 |
| Chronological age | – 0.315 | – 0.003 | – 0.318 |
| Being female | 0.174 | – 0.042 | 0.132 |
| Years of education | 0.110 ** | 0.002 | 0.112 ** |
| Self-rated health | 0.290 | 0.057 | 0.347 |
| Retired | – 0.057 | – 0.144 | – 0.201 |
| Engaged lifestyle | 0.611 | – 0.072 | 0.539 |
|  |  |  |  |
| Cov (*ATOA, Mental felt age)* | – 0.143 ** |  |  |
| Cov (*AARC-Gains, AARC-Losses)* | 0.225 ** |  |  |

Note: AARC: awareness of age-related change; ATOA: attitudes toward own aging.

** p<0.01; * p<0.05
